# Painful Abdominal Scars

**Published:** 1919

**Authors:** 


***Painful Abdominal Scars.*** By Major E. P. Quain and Captain C. Eggers. *The Military Surgeon*, August, 1918.

The authors speak of the pain in and about a scar accompanied by dragging sensations, accumulation of gas and constipation, complained of by a number of patients, and ask: In which cases are the symptoms complained of due to actual pathologic changes, and in which cases are they of neurotic or "slacker" origin?

In the majority of cases, patients were returned to duty after examination; some were operated on (here the authors give in detail 7 cases) and were then sent back to their units.

In most cases observed, abdominal scars were the result of clean or suppurative appendicitis; several patients had been operated on for inguinal hernia and one for duodenal ulcer. The causes producing painful scars may be divided into four groups: (1) Simple adhesions of omentum or gut to the partial peritoneum under and surrounding the scar. (2) Small submuscular hernias of omentum through the peritoneum. (3) Thin, stretched out scars with hernia-like bulging of the abdominal wall, together with numerous adhesions. (4) Retention of appendix and adhesions following drainage of an appendix abscess. To these may be added cases in which nerve fibers have been caught in the scar and produce pain. Excision of the scar would bring about a cure.

In some cases, the conditions found at operation were due to faulty technic at the time of the first operation and might have been prevented; in others, the infection had invaded the tissues and caused such damage to the abdominal wall that permanent pathology was inevitable.

